# Clinical guideline seom: hereditary colorectal cancer

**DOI:** 10.1007/s12094-015-1439-z

**Published:** 2015-11-19

**Authors:** C. Guillén-Ponce, R. Serrano, A. B. Sánchez-Heras, A. Teulé, I. Chirivella, T. Martín, E. Martínez, R. Morales, L. Robles

**Affiliations:** Medical Oncology Department, Hospital Universitario Ramón y Cajal, Carretera de Colmenar Viejo, KM 9,100, 28034 Madrid, Spain; Medical Oncology Department, Hospital Reina Sofía, Córdoba, Spain; Medical Oncology Department, Hospital General Universitario de Elche, Elche (Alicante), Spain; Medical Oncology Department, Instituto Catalán de Oncología (ICO), Barcelona, Spain; Medical Oncology Department, Hospital Clínico Universitario de Valencia, Valencia, Spain; Medical Oncology Department, Hospital Universitario de Salamanca, Salamanca, Spain; Medical Oncology Department, Hospital Marqués de Valdecilla, Santander, Spain; Medical Oncology Department, Hospital La Mancha Centro, Alcázar De San Juan (Ciudad Real), Spain; Medical Oncology Department, Hospital Doce de Octubre, Madrid, Spain

**Keywords:** Hereditary colorectal cancer, Lynch syndrome, Adenomatous polyposis, Colon cancer

## Abstract

Genetic mutations have been identified as the cause of inherited cancer risk in some colon cancer; these mutations are estimated to account for only 5–6 % of colorectal cancer (CRC) cases overall. Up to 25–30 % of patients have a family history of CRC that suggests a hereditary component, common exposures among family members, or a combination of both. Cancers in people with a hereditary predisposition typically occur at an earlier age than in sporadic cases. A predisposition to CRC may include a predisposition to other cancers, such as endometrial cancer. We describe genetics, current diagnosis and management of CRC hereditary syndromes pointing to a multidisciplinary approach to achieve the best results in patients and family outcomes.

## Introduction

In Spain, cancer is the primary cause of death in males (31 %) and the second major cause in women (20 %) after cardiovascular disease. Approximately 5–10 % of cancer has a hereditary component with high penetrance alleles. In addition, up to 25–30 % of certain cancers such as colorectal cancer (CRC) have a familial component due to the inheritance of alleles with a moderate penetrance. It is likely that other undiscovered genes and background genetic factors contribute to the development of familial cancer in conjunction with non-genetic risk factors [1].

Hereditary CRC syndromes caused by known high-penetrance genes collectively account for 5–6 % of all cases of CRC. This group includes hereditary non polyposis colorectal cancer, also known as Lynch Syndrome (LS), adenomatous (familial adenomatous polyposis [FAP] and MUTYH-associated polyposis [MAP]) and hamartomatous (Peutz-Jeghers Syndrome [PJS], Juvenile Polyposis Syndrome [JPS], PTEN-Hamartomatous Tumor Syndrome [PHTS]) polyposis syndromes [2]. The altered genes involved in cancer onset are now well known. Almost all gene mutations known to cause a predisposition to CRC are inherited in an autosomal dominant fashion, although, there is at least one example of autosomal recessive inheritance, such as MAP.

When the family history includes two or more relatives with CRC, the possibility of a genetic syndrome is increased substantially. The first step in this evaluation is a detailed review of the family history to determine the number of relatives affected, their relationship to each other, the age at which the CRC was diagnosed, the presence of multiple primary CRCs, and the presence of any other cancers (e.g., endometrial) consistent with an inherited CRC syndrome. Differential diagnosis is essential for management and cancer prevention of the affected individuals, because each syndrome has its own distinctive organ-specific manifestation and requires a different surveillance strategy. In addition, the genetic diagnosis of hereditary cancer syndromes allows predictive genetic analysis to be performed in at risk family members. Healthy individuals who are carriers of a mutation are at high risk of developing certain diseases, depending upon the syndrome. Both affected patients as well as at risk individuals are offered screening programs pertinent to their situation as well as prophylactic treatment with the aim of reducing risk and detect precursor lesions or cancer at an early stage.

## Methodology

Levels of evidence and recommendations assess the strength of the evidence supporting the use of specific interventions or approaches, based on the following:

Grade A (levels of evidence: Ia, Ib): requires a minimum of a randomized clinical trial that forms part of a larger good quality and consistent clinical trial in terms of the specific recommendations.

Grade B (levels of evidence: IIa, IIb, III): requires the availability of methodologically correct clinical trials that are randomized with regard to the recommendation topic, including trials that do not comply with the criteria A or C.

Grade C (levels of evidence: IV): requires the availability of documents and opinions from expert committees and/or known authorities with clinical experience; indicates the absence of directly applicable and high-quality clinical studies.

## Lynch syndrome (LS) (also called hereditary nonpolyposis colorectal cancer)

### Clinical and molecular diagnosis of LS

LS is an hereditary condition that increases the risk of CRC, endometrial (EC) and other cancer types (ovarian, upper urinary tract, gastric, small intestine, pancreas, biliary tract, gliomas and sebaceous glands). It is an autosomal dominant condition caused by germline mutations in genes involved in the repair of DNA damage during DNA replication [mismatch repair genes (MMR): MLH1, MSH2, MSH6 y PMS2]. Also, LS is caused by deletions of *EPCAM* gene, located just upstream from *MSH2*, through epigenetic silencing. A mutation in one of these genes confirms the diagnosis in the patient and in at-risk family members.

The Amsterdam I-II clinical criteria were established to identify families with LS. The revised Bethesda guidelines, the most used criteria, have a better sensitivity. Computational models have been developed to calculate risk of having an *MLH1*, *MSH2*, *MSH6* gene mutation such as MMRpredict, MMRpro, or PREMM_1,2,6_. When the risk of having a MMR gene mutation calculated by computational models is >5 %, genetic testing must be considered. However, clinical criteria and computational models have no optimal sensitivity and efficiency. Several studies testing all CRCs reveal that up to 28 % of LS patients would be missed with the revised Bethesda guidelines. Universal tumor testing for DNA MMR deficiency of all CCR is cost-effective. Immunohistochemistry (IHC) testing of tumor tissue to detect lack of expression of MMR proteins, that can direct germline testing, has an overall reported sensitivity of 83 % and specificity of 89 %. The accuracy of IHC depends of the experience of the laboratory performing the testing. If IHC cannot be done or the result is ambiguous, microsatellite instability (MSI) analysis (the Bethesda’s consensus panel defined in 1998) should be performed. The sensitivity for MSI is estimated at 85 %, and specificity at 90 %. If a tumor sample is not available, germline testing is reasonable [3–6].

About 10–15 % of sporadic CRC show MSI and loss of MLH1 protein. It can be due to somatic events such as promoter hypermethylation or a *BRAF* mutation, which increases with age. Almost no LS tumors have a *BRAF* mutation [3–7].

**Fig. 1:**
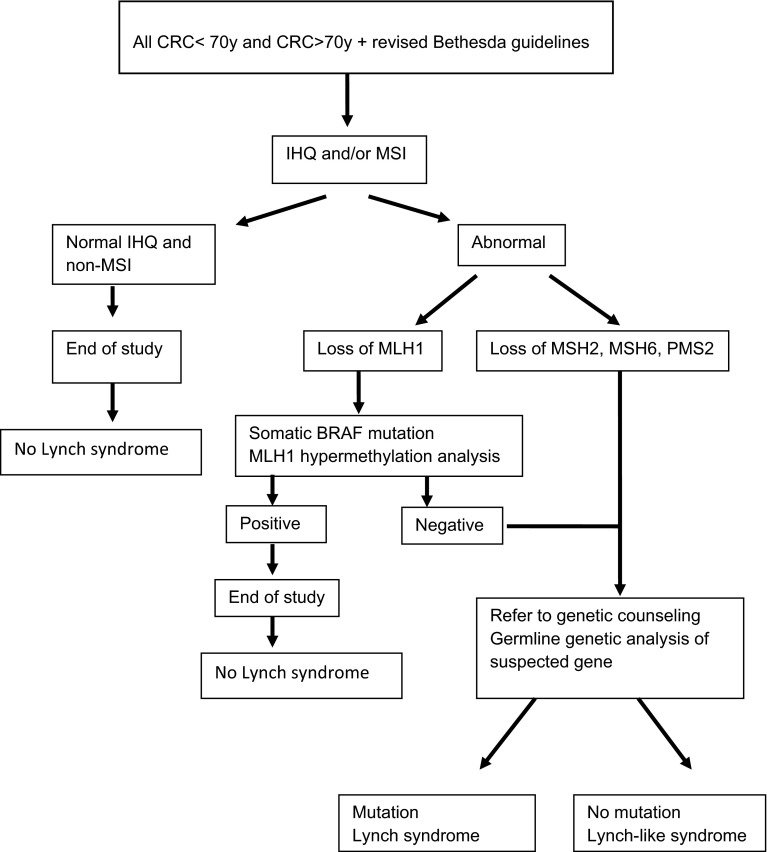
Lynch syndrome: diagnostic algorithm

Lynch-like syndrome defines families with MSI and/or IHC loss of expression of the MMR gene proteins in tumor tissue but no pathogenic germline mutation can be found (Fig. 1).

#### Recommendations

Tumor testing with IHC for MMR proteins and/or MSI of DNA should be assessed in individuals with CRC younger than 70 years old, and in those who fulfill any of the revised Bethesda guidelines (Grade B).

Tumors that demonstrate loss of MLH1 expression should undergo *BRAF* testing or analysis for *MLH1* promoter hypermethylation (Grade B).

Germline testing must be done by DNA sequencing and large rearrangement analysis (multiplex ligation-dependent probe amplification [MLPA]). When the gene mutation is found, mutation-specific germline testing should be done in the at-risk relatives (Grade A).

### Management of LS

#### Screening of associated tumors to LS

LS shows incomplete penetrance and a variable expression. There are some differences in the clinical phenotype of patients with LS depending on the MMR gene mutated. The majority of mutations described in LS are in the *MLH1* and *MSH2* genes. The accumulated risk of CRC up to 70 years varies between 34 and 47 %, for *MLH1* and *MSH2,* respectively. The risk of EC varies between 18 % (*MLH1*) and 30 % (*MSH2*) [8]. The risk of extracolonic cancers as well as multiple tumors seems to be higher in families with an *MSH2* mutation [8]. The mutations in *MSH6* or *PMS2* cause an attenuated phenotype (later age at diagnosis and lower penetrance) [9–11]. The contribution of *MSH6* and *PMS2* to LS is small, although recent population studies suggest that the prevalence in families with a mutation in these genes could be higher than that expected [12].

The precursor lesion for CRC is an adenomatous polyp with high-grade dysplasia. The adenoma-carcinoma sequence is accelerated (estimated at 35 months) compared with 10–15 years in sporadic cancer [13].

Colonoscopy and removal of all polyps has demonstrated a reduction in the incidence of CRC and the mortality of individuals with LS [14].

The screening tests for gynecological cancers have a low sensitivity/specificity [15].

##### Recommendation

Colonoscopy is recommended every 1–2 years starting at age 25 or 5 years younger than the youngest case in the family (if diagnosed before age 25). From age 40 this changes to annual screening. The upper age limit depends upon the health of the patient (Grade B).

In carriers of *MSH6* mutations colonoscopy is recommended every 2–3 years starting at age 30–35 (or 10 years before the age of the youngest case in the family). Whilst in carriers of *PMS2* mutations, screening starts at 35–40 years of age at the same frequency (2–3 years) unless an early-onset cancer exits in a given family (Grade B).

In women with LS, the recommended screening protocol is a gynecological examination; transvaginal ultrasonography with endometrial aspiration and optional CA-125 tumor marker detection is recommended starting at 30–35 years of age and performed every year (Grade C).

In families with aggregation of gastric cancer or urothelial tumors an upper endoscopy with duodenoscopy every 3–5 years, starting at 30–35 years of age and/or cytological analysis of urine and renal ultrasonography every 1–2 years, starting at 25–30 years of age (Grade C).

It has been suggested that families with LS have an increased risk of breast cancer but there is insufficient evidence to give specific screening recommendations. Nor is there sufficient evidence for screening of other tumors associated with LS (brain, pancreas, biliary tract, small bowel, etc.).

#### Risk reducing surgery

A decision analytic model designed to compare annual gynecologic examinations with annual screening (ultrasonography, endometrial biopsy, CA 125) and with hysterectomy with bilateral salpingo-oophorectomy at age 30 years in LS women demonstrated that surgical management led to the greatest expected life years, and when comparing prophylactic surgery with the screening option, one would need to perform 75 surgeries to save one woman’s entire life. For cancer prevention, however, only 28 and 6 prophylactic surgeries would need to be performed to prevent one case of ovarian and endometrial cancer, respectively [15].

Schmeler et al. showed that in mutation-positive women prophylactic hysterectomy with bilateral salpingo-oophorectomy was an effective strategy for preventing endometrial and ovarian cancer in women with the Lynch syndrome [16].

##### Recommendation

Female mutation carriers that no-longer want to have children can be offered the possibility of a hysterectomy and bilateral salpingo-oophorectomy, especially in carriers of a MSH6 mutation (Grade A).

There are no sufficient data to recommend prophylactic colectomy in individuals at risk or mutation carriers (Grade C).

#### Chemoprevention

In the CAPP2 study, 861 patients diagnosed with LS were randomized to receive aspirin (AAS): 600 mg every 24 h versus placebo. Those patients that received AAS for more than 2 years had a 54 % reduction in the incidence of CRC (hazard ratio [HR]: 0.41, confidence interval [IC] 95 %: 0.19–0.86, *p* = 0.02) and a reduction in the incidence of other tumors (HR: 0.47, IC95 %: 0.21–1.06, *p* = 0.07). In those patients that received AAS for less than 2 years there was no CRC reduction benefit [17–19].

##### Recommendation

Systematic administration of aspirin is not recommended to prevent CCR in patients with LS (Grade C).

## Familial adenomatous polyposis (fap)

### Clinical and molecular diagnosis of FAP: classical y attenuated forms

FAP, also known as *APC*-Associated Polyposis, is an inherited autosomal dominant syndrome characterized by the development of hundreds of colorectal adenomas in the second or third decade of life. It is the most common gastrointestinal polyposis syndrome (1 per 10.000 subjects). If patients are not treated, all will develop CRC at an average age of 38–41 years [20]. It represents less than 1 % of all CRC cases. Extracolonic manifestations are gastric and duodenal polyps (duodenal adenomas are found in more than 80 % of patients, but the risk of developing a duodenal cancer is less than 15 %) [21], desmoids tumors, thyroidal, liver (hepatoblastoma) and brain tumors, osteomas, congenital hypertrophy of retinal pigmented epithelium, supernumerary teeth, epidermoid cysts and adrenal masses.

APC is a tumor-suppressor gene located on chromosome 5q that plays an important role in the *Wnt* signaling pathway by negatively regulating the *ß*-catenin oncoprotein. Germline mutations in *APC* gene are responsible for FAP. Mutational hotspots in this gene are located in the 5′ region of exon 15. Most of the germline mutations are inherited but between 11 and 25 % of cases can be de novo.

Attenuated FAP (AFAP) is a phenotypic variant of FAP characterized by a mild disease course, a reduced number of colorectal adenomas (10–99) with a right-sided distribution in the colon, later age of onset a and lower CRC risk (up to 70 %) if patients are not treated in a timely manner [22].

A genotype-phenotype correlation has been described. An aggressive polyposis phenotype is associated with mutations from codons 1250 to 1464; otherwise, AFAP is associated with mutations located at either end of the gene or in exon 9.

#### Recommendation

Patients with more than 100 colonic adenomas should be tested for an *APC* gene mutation. If no mutation is detected, the *MUTYH* gene should be analyzed (Grade B).

When PAFA is suspected, both *APC* and *MUTYH* genes could be analyzed. On the one hand, if autosomal dominant inheritance is observed, the *APC* gene should be tested first, whereas if recessive inheritance is observed, testing for *MUTYH* should be done first (Grade B).

*POLE* and *POLD1* genes could be evaluated for testing if no mutation detected in *APC* and *MUTYH* and clinically suspected (Grade B).

Massive parallel sequencing will probably change this sequential approach.

### Management of FAP and AFAP

#### Surgical options in FAP and AFAP

Clinical diagnosis of FAP is based on the presence of ≥100 polyps, and the lifetime risk to develop a cancer is 100 % at around 50 years of age. Surgery is the most important preventive measure in patients with FAP [23].

Surgical options in FAP patients are total abdominal colectomy with ileorectal anastomosis (TAC/IRA) or total protocolectomy with pouch anal anastomosis (TPC/IPAA).

If AFAP presents with fewer than 100 polyps, colectomy is usually not necessary [24].

##### Recommendation

For patients with FAP, most frequently, after puberty has been reached or when adequate polyp control cannot be achieved by endoscopic technique, colectomy should be performed (except in those cases where the size or histology recommends surgery earlier) (Grade C).

#### Surveillance in FAP and AFAP

Colonic surveillance

Classical FAP

The surveillance is based on colon cancer risk, median age of diagnosis and/or surgery performed.

##### Recommendation

In classical FAP, preoperative surveillance (Grade B):

- Individuals with a family history and no familial mutation found:
  - Biennial flexible sigmoidoscopy beginning from the age of 10 to 15 years until 40 years. Every 3–5 years until age 50 and from then on less frequently. If new polyps are detected, a total colonoscopy should be done; both the follow up and treatment will be the same as a regular patient [25].
- Individuals with a positive genetic test:
  - Biennial flexible sigmoidoscopy beginning from the age of 10 to 15 years. Once adenomatous polyps have been identified, colonoscopy should be performed on an annual basis until the patient undergoes surgery [25].

Surveillance after colectomy (Grade B):

- In TAC/IRA patients, proctoscopy every 6–12 months is recommended [25].
- In TPC/IPAA patients, ileoscopy is recommended every 1–3 years, depending on the detection of adenomatous transformation [25].

AFAP

Average age of cancer development is 55 years; diagnosis before 20 years is extremely unusual.

##### Recommendation

Biennial colonoscopy should be performed beginning from 18 to 20 years (Grade B).

Extracolonic surveillance

- Upper gastrointestinal tract.

Duodenal polyps are found frequently (around 50–90 %).

Duodenoscopic findings are assessed using the Spigelman classification (which describes 5 stages assessing the polyp number, size, histology and type of dysplasia).

Duodenal cancer risk is 5 %, increasing to 36 % in patients with Spigelman stage III–IV [25].

##### Recommendation

Recommendations are different depending on the stage (duodenoscopic from 5 years in stage 0/I to propose surgery in stage IV). Upper endoscopy and Vater´s ampoule endoscopy should begin between 25 and 30 years.

- Desmoids tumors:

Approximately 10–15 % of mutation carriers will develop a desmoids tumor, usually intra-abdominal. Risk factors are abdominal surgery, family history and mutation in 1444 codon [25].

##### Recommendation

Computed tomography (CT) and magnetic resonance imaging (MRI) are useful when a desmoids tumor is suspected.

Non-steroidal anti-inflammatory drugs (NSAIDs) vs sulinac with tamoxifen are recommended (Grade B). After progression chemotherapy should be used: dacarbazine, methotrexate, vinblastine or radiotherapy. Surgery is controversial and should be reserved for abdominal complications (bowel obstruction, intestinal ischemia) (Grade B).

- Thyroid:

Patients with classical FAP have a lifetime thyroid cancer risk of 2–6 % and female predominance (95 %). Peak of incidence is in the third decade of life [25].

##### Recommendation

Annual thyroid physical examination and ultrasound are recommended beginning from the age of 15 years (Grade C).

- Hepatoblastoma

The risk for hepatoblastoma in FAP is 750 to 7500 times higher than in the general population, although the absolute risk is estimated at less than 2 %. The majority of hepatoblastomas occur prior to the age of 3 years [26].

##### Recommendation

Screening for hepatoblastoma in FAP using frequent (every 2–3 months) abdominal ultrasound examinations and measurement of serum alpha-fetoprotein concentrations may be considered from infancy to the age of 5 years. However, the optimal interval for hepatoblastoma screening in FAP is not known (Grade C).

Chemoprevention in FAP and AFAP

Cyclooxygenase-2 (COX-2) has been shown to be over-expressed in colorectal adenomatous polyps and cancers. NSAIDs have been shown to reduce the incidence and recurrence of colorectal adenomatous polyps [25].

##### Recommendation

Sulindac and celecoxib have an additional role to surgery; nonetheless, they should never replace surgery and should not be recommended in those patients with any cardiovascular disease (Grade C). It is also important to continue an endoscopic surveillance in patients with residual polyps.

## *Mutyh*-associated polyposis (map)

### Clinical and molecular diagnosis of MAP

MAP is caused by biallelic mutations in *MUTYH* that is characterized by an increased lifetime risk of CRC (43 % to almost 100 %). MAP is suspected in an individual who has: colonic adenoma count between one and ten before the age of 40; colonic adenoma and/or hyperplastic polyp count between ten and a few hundred; colonic polyposis (i.e., >100 colonic polyps) in the absence of a germline *APC* mutation; CRC with the somatic *KRAS* mutation c.34G > T in codon 12; and, family history of CRC (with or without polyps) consistent with autosomal recessive inheritance [27].

The diagnosis is determined in individuals with characteristic clinical findings and biallelic *MUTYH* mutations [28]. Two common mutations, c.536A > G (p.Tyr179Cys) in exon 7 and c.1187G > A(p.Gly396Asp) in exon 13, account for up to 70 % of persons with MAP [29].

#### Recommendation

Offer molecular genetic testing for the familial mutations to all siblings of an individual with genetically confirmed MAP to reduce morbidity and mortality through early diagnosis and treatment (Grade A).

### Management of MAP

Typically associated with ten to a few hundred colonic adenomatous polyps that are evident at a mean age of about 50 years, colonic cancer develops in some individuals with biallelic *MUTYH* mutations in the absence of polyposis [30]. Duodenal adenomas are found in 17–25 % of individuals with MAP; the lifetime risk of duodenal cancer is about 4 %. Also there is a modestly increased risk for other malignancies of the ovary, bladder, and skin, and some evidence for an increased risk for breast and EC [31]. More recently, thyroid abnormalities (multinodular goiter, single nodules and papillary thyroid cancer) have been reported in some studies. Some affected individuals develop sebaceous gland tumors.

Available data suggest that heterozygous relatives of patients with MAP have a two- or at most threefold increase in their risk for colorectal cancer at an age similar to that in the general population [32].

#### Recommendation

Suspicious polyps identified on colonoscopy should be removed until polypectomy alone cannot manage the large size and density of the polyps, at which point either subtotal colectomy or proctocolectomy is performed (Grade A). Duodenal polyps showing dysplasia or villous changes should be excised during endoscopy (Grade A).

Abnormal findings on thyroid ultrasound examination should be evaluated by a thyroid specialist to determine what combination of monitoring, surgery, and/or fine needle aspiration (FNA) is appropriate (Grade C).

Surveillance for individuals with biallelic *MUTYH* germline mutations should be performed by pancolonoscopy beginning at age 18–20 years; upper endoscopy with side viewing duodenoscopy beginning at age 25–30 years; follow-up depending on disease severity (Grade A).

There are no specific screening recommendations for individuals heterozygous for a *MUTYH* mutation. They are expected to benefit from population screening measures or could be offered average moderate-risk colorectal screening based on family history (Grade C).

## Peutz-jeghers syndrome (pjs)

### Clinical diagnosis and genetic testing

PJS is an autosomal dominant hereditary condition characterized by the association of hamartomatous gastrointestinal polyposis, typical mucocutaneous pigmentation and cancer predisposition. Peutz-Jeghers polyps are more common in the small intestine but can also occur in the stomach, large bowel and extra-intestinal sites. Hyper-pigmentation is typically present in childhood around the mouth, eyes, nostrils, oral mucosa and fingers and may fade in puberty and adulthood. Individuals with PJS are at increased risk for a wide variety of cancers: epithelial malignancies (colorectal, gastric, pancreatic, breast and ovarian), adenoma malignum of the cervix (females) and some benign male and female genital tumors.

Clinical diagnosis of PJS is based on any of the following criteria [33]:

- Two or more histologically confirmed PJS-type hamartomatous polyps.
- Any number of PJS-type polyps detected in one individual who has a family history of PJS in a close relative.
- Characteristic mucocutaneous pigmentation in an individual who has a family history of PJS in a close relative.
- Any number of PJS-type polyps in an individual who also has characteristic mucocutaneous pigmentation.

Genetic testing of *STK11/LKB1* gene can find pathogenic variants in 80–94 % of the affected individuals. Approximately 45 % of affected individuals have no family history.

### Management of PJS

Upper and lower endoscopies may allow early detection of colorectal and upper gastrointestinal cancers. Colorectal cancer risk has been reported from age 27 to 71 with 39 % lifetime risk estimates. Lower estimates have been described for upper gastrointestinal cancer [34].

The other indication for endoscopic surveillance is early detection and prevention of polyp related complications.

Cumulative breast cancer risk estimates range from 31 to 54 % at age 60, with a mean age at diagnosis of 37 [34, 35].

#### Recommendation

Endoscopic surveillance from age 8 including upper endoscopy and video capsule endoscopy or magnetic resonance enterography and colonoscopy may allow early diagnosis of polyposis and appropriate treatment. If a significant polyposis is found, endoscopy should be repeated every three years: if not then from age 18 and every three years. At age 50 the endoscopy should be performed every 1–2 years (Grade C)

Annual breast MRI examinations in females aged 25–30 and mammograms from age 50 are recommended. Routine pap smears from age 25 every 2–3 years using liquid based cytology are also recommended (Grade C).

Regular testicular examinations and testicular ultrasound in the case of abnormal findings is recommended from the pediatric age (Grade C).

## Juvenile polyposis syndrome (jps)

### Clinical diagnosis and genetic testing

JPS is characterized by predisposition to specific hamartomatous polyps (“juvenile polyps”) in the stomach, small intestine, colon and rectum. Most juvenile polyps are benign and may cause anemia and bleeding. Malignant transformation of the gastrointestinal tract is mainly due to colon cancer but also gastric, upper GI tract and pancreatic cancer have been reported.

Any of the three following criteria is clinical diagnostic of Juvenile Polyposis Syndrome:

- 5 or more juvenile polyps of the colorectum.
- Multiple juvenile polyps throughout the GI tract.
- Presence of any number of juvenile polyps and family history of JPS.

Approximately 20 % of individuals have pathogenic variants in *BMPR1A* and 20 % in *SMAD4*. Most individuals with *SMAD4* variants may present with a combined syndrome: JPS and hereditary hemorrhagic telangiectasia. In 25 % of probands with JPS there is no family history and they may harbor de novo mutations [36].

### Management of JPS

Upper and lower endoscopies may allow early detection of CRC and upper gastrointestinal cancers. In large kindreds, the CRC risk estimate was 38 % and upper gastrointestinal cancer 20 %. The youngest CRC diagnosis was at age 17 and the youngest upper gastrointestinal cancer was at age 20 [37].

#### Recommendation

Colonoscopy and upper GI endoscopy beginning at age 15 and at least every 3 years and appropriate polypectomy or surgery if needed is recommended (Grade C).

## Serrated polyposis syndrome (sps)

### Clinical and genetic diagnostics

SPS is characterized by the presence of multiple serrated polyps and/or of a large size in the colon that predisposes an increased risk to develop CRC [38].

A clear genetic etiology is not confirmed. In presence of concurrent adenomas the study of *MUTYH* mutations may be done [39].

The management is empirical and includes the resection of the polyps, endoscopic surveillance and genetic counseling. A colectomy with IRA is recommended before discovering a CRC.

#### Recommendation

Complete resection and endoscopic surveillance every 5 years from 35 to 40 years of age (or 10 years before the youngest case in the family).

With regard to the risk of CRC in first‐degree relatives, surveillance colonoscopy is recommended every 5 years from 35 to 40 years of age (or 10 years before the youngest case in the family), modifying the protocol if polyps are found (Grade B).

## Hamartomatosis syndrome associated with pten (phts)/cowden syndrome

### Clinical and genetic diagnostics of PHTS

PHTS is characterized by muco-cutaneous lesions of a papillomatosis form on the face and mouth with a typical cobbled appearance, acral keratosis, macrocephaly, the development of a dysplastic gangliocytoma of the cerebellum, thyroid and breast lesions, gastrointestinal polyps or uterine leiomyomas [40]. The risk of developing breast cancer is from 25 to 50 %, thyroid cancer from 3 to 10 % and EC from 5 to 10 %. Up to 92 % of the cases of Cowden have polyps and the prevalence of CRC is from 9 to 18 % [41].

It is inherited in an autosomal dominant manner and forms part of the clinical spectrum of diseases associated with a germline mutation in *PTEN* [42].

### Management of PHTS

There is no curative treatment for these syndromes, although muco-cutaneous lesions should be treated by surgery (curettage or cryosurgery), as they are not usually resolved with topical treatment. The facial tricholemmomas respond to the usual laser treatment.

There are no data that support the efficacy of prophylactic surgery with regard to the reduction in the risk of cancer or the mortality impact.

#### Recommendation

The recommendations include breast, thyroid and skin surveillance, basal colonoscopy basal at 50 years of age and screening for endometrial cancer (Grade B).

## Other genes involved in genetic predisposition to crc (polymerase proofreading-associated syndrome)

Germ-line mutations in the exonuclease (proofreading) domain of DNA polymerase *POLE* and *POLD1* have been recently associated with a dominantly inherited syndrome that confers increased risk to polyposis and CRC.

These genes have been studied by a Spanish group in 529 kindred (441 with familial nonpolyposis CRC and 88 with polyposis) using massively parallel sequencing identifying 7 genetic variants (the *POLE* p.L424V mutation was associated with polyposis, CRC and oligodendroglioma) and 6 *POLD1* variants with strong evidence for pathogenicity were identified in nonpolyposis CRC families [43].

*POLD1* mutations have been associated with EC and breast tumors, as well.

**Fig. 2:**
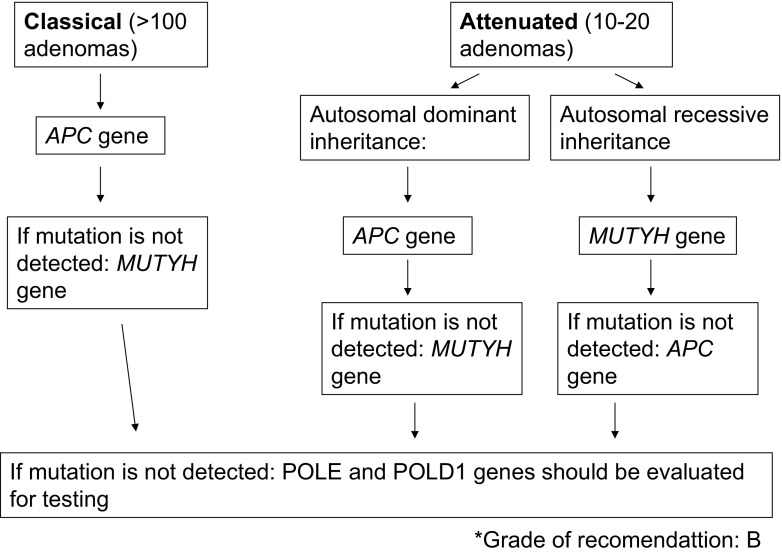
Adenomatous polyposis: diagnostic algorithm. Grade of recommendation: B

Therefore, the genetic testing of these two genes should be recommended if a clinical phenotype is suspected (Fig. 2).
